# Biochemical characterization of predicted Precambrian RuBisCO

**DOI:** 10.1038/ncomms10382

**Published:** 2016-01-21

**Authors:** Patrick M. Shih, Alessandro Occhialini, Jeffrey C. Cameron, P John Andralojc, Martin A. J. Parry, Cheryl A. Kerfeld

**Affiliations:** 1 Department of Plant and Microbial Biology, University of California, Berkeley, California 94720-3102, USA; 2 Department of Plant Biology and Crop Science, Rothamsted Research, Harpenden, Herts AL5 2JQ, UK; 3 Lancaster Environment Centre, Lancaster University, Bailrigg, Lancaster LA1, 4YQ, UK; 4 Department of Biochemistry and Molecular Biology, DOE Plant Research Laboratories, Michigan State University, East Lansing, Michigan 488242, USA; 5 Physical Biosciences Division, Lawrence Berkeley National Laboratory, Berkeley, California 94720, USA

## Abstract

The antiquity and global abundance of the enzyme, RuBisCO, attests to the crucial and longstanding role it has played in the biogeochemical cycles of Earth over billions of years. The counterproductive oxygenase activity of RuBisCO has persisted over billions of years of evolution, despite its competition with the carboxylase activity necessary for carbon fixation, yet hypotheses regarding the selective pressures governing RuBisCO evolution have been limited to speculation. Here we report the resurrection and biochemical characterization of ancestral RuBisCOs, dating back to over one billion years ago (Gyr ago). Our findings provide an ancient point of reference revealing divergent evolutionary paths taken by eukaryotic homologues towards improved specificity for CO_2_, versus the evolutionary emphasis on increased rates of carboxylation observed in bacterial homologues. Consistent with these distinctions, *in vivo* analysis reveals the propensity of ancestral RuBisCO to be encapsulated into modern-day carboxysomes, bacterial organelles central to the cyanobacterial CO_2_ concentrating mechanism.

## Abstract (web summary)

The enzyme RuBisCO has evolved over billions of years and catalyses reactions in plants and bacteria, although why some reactions persist is unclear. Here, the authors resurrect ancestral RuBisCO to reveal aspects of the Precambrian atmosphere and the selective pressures governing RuBisCO evolution.

## Graphical abstract

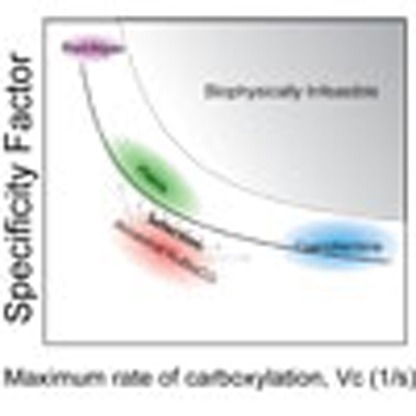

Much of our understanding of Precambrian (0.542–4  Gyr ago) environments is limited to inferences gained from geological proxies. However, the inherent property of evolution in biological systems offers a means by which ancient proteins can be probed to gain fresh insight into the corresponding palaeoenvironments^1^. Using a combination of phylogenetic and molecular biology techniques, it is possible to predict and ‘resurrect' ancient proteins with ancestral sequence reconstruction methods and gene synthesis. One particular enzyme that has had profound implications for life on Earth is RuBisCO, the predominant enzyme responsible for carbon fixation, whose activity over billions of years has linked biological systems to their inorganic environment through global biogeochemical cycles.

Form 1 RuBisCO is composed of eight large (LSUs) and eight small (SSUs) subunits, forming a hexadecameric (L_8_S_8_) holoenzyme. RuBisCO exhibits dual substrate specificity (CO_2_ versus O_2_). Carboxylation of the acceptor molecule, ribulose-1,5-bisphosphate (RuBP) generates two molecules of 3-phosphoglycerate that are used in the regeneration of RuBP and synthesis of biomolecules. Oxygenation of RuBP generates one molecule of 3-phosphoglycerate and one molecule of 2-phosphoglycolate, a toxic metabolite that is catabolized through photorespiration. Loss of CO_2_ and energy through photorespiration decreases the efficiency of carbon fixation, yet the oxygenase activity of RuBisCO has persisted across all known forms of RuBisCO^2^.

Although there are multiple phylogenetically distinct clades of RuBisCO, most studies have been focused on Form 1 RuBisCO. Plants and the majority of cyanobacteria contain the prevalent and well-characterized Form 1B RuBisCO, whereas proteobacteria and a subgroup of marine cyanobacteria contain the sister clade, Form 1A RuBisCO (Fig. 1). While the dating of this divergence event is imprecise, it most likely occurred more than a billion years ago, given that it predates the origins of plastid-containing eukaryotes (Archaeplastida), which fossil records and molecular clock studies have consistently dated between 0.9–1.8 Gyr ago^3^,^4^,^5^,^6^,^7^.

On the basis of previous studies^8^,^9^,^10^ on the decreasing CO_2_ and increasing O_2_ atmospheric concentrations over Earth's history (Fig. 1), changes in the proportions of these two gases have been suggested to be the major evolutionary force driving RuBisCO activity towards higher carboxylation activity^11^. After the great oxidation event (2.5 Gyr ago), the vast majority of the Proterozoic Era experienced relatively low O_2_ concentrations, 5–18% of the present atmospheric levels (PALs)^10^. Moreover, CO_2_ concentrations have been predicted to be between 300 and 600 times higher than PALs based on solar luminosity models^9^. It is important to note that there is still a great deal of controversy surrounding retrodiction of the atmospheric composition of early Earth, as other lines of evidence have challenged the high CO_2_ levels originally predicted by solar luminosity models^12^,^13^. However, these studies have been further questioned owing to the substantial body of literature documenting compelling evidence to support the ‘faint young Sun paradox'^14^,^15^,^16^ or directly by carbon isotope ratios of Proterozoic microfossils^17^. Taken together, selective pressure to improve carboxylase activity may not have occurred until the late Neoproterozoic to early Phanerozoic in response to two dramatic atmospheric changes: (1) a second significant increase in O_2_ levels inferred from sulfur isotope studies^18^ and modelled from the fossil record of charcoal^19^,^20^,^21^; and (2) a decrease in CO_2_ levels to PALs at the start of the Phanerozoic, suggested primarily by solar luminosity models^22^. These changes eventually brought atmospheric levels of CO_2_ and O_2_ to near present-day levels.

In this study, we reconstructed ancestral versions of RuBisCO based on phylogenetic analyses. The predicted protein sequences were then resurrected using gene synthesis to probe the biochemical properties of these ancient enzymes. We also expressed ancestral LSUs in an extant cyanobacterial strain to determine whether they could be incorporated into the carboxysome, the core component of the cyanobacterial carbon concentrating mechanism (CCM) that evolved in response to the changing atmosphere and is essential for survival at current CO_2_ levels. Our findings are presented in the context of the Precambrian palaeoatmosphere.

## Results

### Ancestral sequence reconstruction of Form 1A and Form 1B RuBisCO

For both the LSU and SSU, encoded by the *rbcL* and *rbcS* genes, respectively, we reconstructed the most recent common ancestor (MRCA) of the Form 1A clade (α-MRCA), the Form 1B clade (β-MRCA) and both Form 1A and 1B clades (α/β-MRCA) (Figs 2 and 3). The ancestral proteins were predicted from independently derived phylogenetic trees for RbcL and RbcS containing a broad diversity of Form 1A and 1B RuBisCO (>100 sequences; Figs 4 and 5; Supplementary Tables 1 and 2). Maximum-likelihood algorithms^23^ were used to reconstruct the most probable ancestral sequence for each ancestral node, as well as for determining the posterior probability for each amino acid position (Supplementary Fig. 1). The amino acid substitutions between the ancestral and extant counterparts are distributed across the entirety of both the LSU and SSU, not focused in any one region of the primary structure (Figs 2, 3 and 6). It is important to note that given the antiquity of these sequences, even the best predictions will probably contain imprecision^24^, as there are various positions in RbcS that have low posterior probabilities. Despite the possibility that these potentially incorrectly predicted sites would result in misfolded protein, we proceeded in producing the proteins as the synthesized sequences were the most probable and presented an opportunity to characterize the palaeoenzymatic properties of the predicted ancestral enzymes. The origin of these ancestral proteins can most reliably be traced back to the Mesoproterozoic Era or earlier, given that they must have existed before the plastid endosymbiosis event.

### Biochemical characterization of ancestral RuBisCO

To investigate the biochemical properties of ancestral RuBisCO, the genes were synthesized, expressed in *Escherichia coli* (*E. coli*), and the resulting proteins subsequently purified (further described in the Methods section). In addition, Form 1A and Form 1B bacterial RuBisCOs from *Prochlorococcus marinus* MIT9313 and *Synechococcus* sp. PCC6301, were heterologously expressed, purified and assayed, to provide extant enzyme comparisons. Before this study, only one Form 1A RuBisCO had ever been fully characterized (from *Chromatium vinosum*)^25^; thus, the full biochemical characterization of the *Prochlorococcus* RuBisCO provides much needed additional data on Form 1A RuBisCOs. Although most ancestral sequence reconstruction studies have focused primarily on monomeric proteins owing to the potential challenges of reconstituting ancestral protein–protein interactions, we were able to purify ancestral RuBisCO enzymes from the β-MRCA and α-MRCA. Soluble α/β-MRCA enzyme could not be recovered, possibly due to misfolding or misprediction of the ancestral sequence; this is not surprising owing to the fact that this is the most remote of the three ancestral variants synthesized. Remarkably, purified 520 kiloDalton hexadecameric L_8_S_8_ β-MRCA and α-MRCA holocomplexes were fully assembled as determined by size-exclusion chromatography (Supplementary Fig. 2) and were enzymatically active (Table 1).

Owing to the dual carboxylase and oxygenase activities of RuBisCO, there are many catalytic properties that can be both measured and derived. One key parameter in interpreting the efficiency of any given RuBisCO is the *V*_c_, which represents the carboxylation turnover rate under substrate-saturated conditions. Biochemical analysis of ancestral RuBisCOs indicates that the β-MRCA and α-MRCA displayed lower *V*_c_ than those of extant bacterial RuBisCOs (Table 1). This intuitively makes sense, as the Proterozoic atmosphere would have provided little selective pressure to drive RuBisCO to evolve improved kinetic properties, given that the CO_2_/O_2_ ratios were orders of magnitude larger than PAL. To more thoroughly compare the properties of ancestral and extant RuBisCOs, the *V*_c_ was normalized to the Michaelis constant for CO_2_ in normal air at current atmospheric levels of O_2_ (*K*_c_^air^). This value (*V*_c_/*K*_c_^air^) describes the initial response of the rate of carboxylation to the CO_2_ concentration, and thus expresses the ability of RuBisCO to function at low CO_2_ concentrations^26^. Consistently, the *V*_c_/*K*_c_^air^ of ancestral RuBisCOs (28.0–37.4 s^−1^mM^−1^) is lower than those of extant eukaryotic RuBisCOs, which range between 53 and 217 s^−1^mM^−1^ (refs 26, 27; Table 1). One notable exception is that of Form 1A RuBisCO from *Prochlorococcus*, which has a lower *V*_c_/*K*_c_^air^ value than the ancestral RuBisCOs, possibly reflecting an evolutionary relationship to its extant form in the α-carboxysome/CCM. This may be further explained by the avid CCM that has been observed in *Prochlorococcus* spp., even when compared with other CCM-containing marine algae in similar environments^28^. It is difficult to draw firm conclusions from the *V*_c_/*K*_c_^air^ values, as this value is contingent on many other factors (that is, ecological niche, physiology, presence/absence of CCMs, and so on) that cannot be known precisely for the ancestral species. Although it is likely to be overly simplistic to focus on atmospheric global averages, it is tempting to speculate that given the historical placement of our ancestral RuBisCOs in the geological history, the high CO_2_ concentrations of the Proterozoic atmosphere would have been necessary for the ancestral enzymes to function adequately. This is of course assuming that ancestral autotrophs were living in environments similar to those of their extant counterparts. This assumption may be reasonable for the β-MRCA, as it represents the common ancestor to all β-cyanobacteria, which occupy a range of varying niches, and yet have retained many common features such as the major components of the β-carboxysome. Speculation on the ecophysiological context for the α-MRCA RuBisCO is more difficult, given that Form 1A RuBisCO can be found across different bacterial phyla.

Although a high *V*_c_ indicates a rapid rate of carboxylation, the specificity—which is the ratio of the maximum rate of carboxylation and the *K*_M_ for CO_2_ (that is, *V*_c_/*K*_c_)—is an equally important parameter when interpreting the overall carboxylation efficiency of any given RuBisCO. The specificity for carboxylation relative to oxygenation [(*V*_c_/*K*_c_)/(*V*_o_/*K*_o_)] is known as the specificity factor. Extensive comparisons of RuBisCO properties between diverse species have shown that *V*_c_ is inversely proportional to specificity factor^29^,^30^,^31^. Therefore, forms of RuBisCO with a relatively low rate of turnover benefit from a relatively high specificity factor. Importantly, the composition of the Proterozoic atmosphere would not have provided any selective pressure to evolve higher specificity factors and thus to improve discrimination between CO_2_ and O_2_. Interestingly, purified β-MRCA and α-MRCA enzymes display specificity factors similar to those of extant bacterial RuBisCOs (∼50; Table 1). This is a relatively low value in comparison with other extant eukaryotic RuBisCOs; values of 70–100 are typical for higher plants (Supplementary Table 3). In response to the significant Neoproterozoic atmospheric changes, bacterial Form 1A and 1B RuBisCOs appear to have followed an evolutionary path which maintained a low specificity factor, but an increased *V*_c_, facilitated by the evolution of the CCMs that effectively increased the concentration of CO_2_ at the active site of RuBisCO to levels at which a higher specificity factor would not be required. In contrast, eukaryotic RuBisCOs diverged and continued along a separate evolutionary path toward improving substrate specificity rather than *V*_c_ (Fig. 7), to the point where *V*_c_ values were compromised to improve specificity factor values, perhaps reflecting their less robust (if any) CCMs relative to those of cyanobacteria.

Considering the protein fitness landscape of RuBisCO, it has been hypothesized that the best-fit curve encompassing the inverse relationship between *V*_c_ and specificity factor defines the upper limit of RuBisCO activity, which is constrained by the physicochemical and structural properties of the enzyme^32^. Although there are a multitude of kinetic parameters, and thus the fitness landscape is highly multidimensional, the two parameters, *V*_c_ and specificity factor, encompass a large portion of the biological relevance of the kinetic tradeoffs of RuBisCO. The two traits are not independent of one another owing to the hypothesized physicochemical tradeoffs (the penalty of higher specificity factor being lower rates of product release and hence diminished rates of carboxylation). Interestingly, ancestral RuBisCOs display relatively low *V*_c_ and specificity when compared with their extant counterparts (Fig. 7a).

It has been proposed that the first RuBisCO was a suboptimal enzyme, but that over time evolutionary pressure improved its kinetic properties^32^. On the basis of the geological record, one may logically assume that the early Neoproterozoic RuBisCO would not yet have been ‘optimized,' as the enzyme would never have existed in an atmosphere that would have provided the necessary selection pressure for increasing specificity. However, it is equally important to consider the environmental niche and physiology of the ancestral organisms. Although tied to the atmospheric composition, selective pressures to optimize RuBisCO may have occurred as early as the origins of oxygenic photosynthesis, as the local oxygen concentration within a cell producing oxygen from photosynthesis would be much higher than in the Proterozoic atmosphere. Moreover, CO_2_ concentrations may be lower in various niches; for example, marine environments may have lower CO_2_ concentrations due to higher pH values than in freshwater. Other potential factors that may contribute to decreased CO_2_ concentrations include increased microbial growth and CO_2_ fixation rates in a given ecosystem. Taken together, it is difficult to confidently interpret the atmospheric composition on geological timescales, and may be even more difficult to speculate on ancestral microbial physiology based on our limited sampling of extant organisms. Nevertheless, there are two hypotheses that logically follow from taking the geological evidence of the Proterozoic atmosphere at face value. First, early Proterozoic RuBisCO started with both low *V*_c_ and specificity and subsequently evolved to optimize the tradeoff between these two parameters. Second, early RuBisCO began with a high *V*_c_ similar to extant bacterial *V*_c_ and subsequently evolved alternative tradeoffs between catalytic rate and substrate affinity. Our biochemical data support the former scenario, due to the position occupied by the ancestral RuBisCOs in a comparative plot of *V*_c_ and specificity factor (Fig. 7b), suggesting that the changing atmospheric conditions provided the selective pressure needed to drive divergent RuBisCO lineages towards the upper limit of carboxylase activity, given the tradeoff between *V*_c_ and specificity factor (Fig. 7b).

It should be emphasized that although these are the sequences predicted to be most probable given the phylogenetic data, there are still sites with low posterior probabilities, which could explain the relatively low values of *V*_c_. Given the inherent potential imprecisions of ancestral sequence reconstruction, there is always the possibility that inaccuracies in the predicted sequences may have affected the biochemical results. Even if the predictions for some of the sites were inaccurate, it is apparent that both ancestral RuBisCOs lie within the same biochemical space. Moreover, they do not reside far from the predicted best-fit curve. In the absence of kinetic characterization of all bacterial Form 1A RuBisCOs, we make the assumption that all extant bacterial Form 1A RuBisCOs are similar, based on the only two bacterial Form 1 RuBisCOs for which both *V*_c_ and specificity factor have been determined (before this study, only the *Chromatium vinosum* RuBisCO had been fully characterized). When plotting the *V*_c_ and specificity factor of both extant *Prochlorococcus* and *Synechococcus* RuBisCOs, the data points were in strong agreement with the best-fit curve describing all extant characterized RuBisCOs (Fig. 2a). Future characterizations will be necessary to make a more conclusive statement about the relationship between ancestral enzymes and their modern counterparts, as the discovery of any extant bacterial RuBisCOs that have a comparably low specificity factor, along with a low *V*_c_—similar to our ancestral RuBisCO properties—would reveal a much larger array of tradeoffs between the two parameters which, nonetheless, do not preclude sustaining growth in the bacterial species.

Corroborating the accuracy of the primary structures of our ancestral RuBisCO is their ability to fold and reconstitute functional hexadecameric holoenzymes; this provides some evidence that our predicted sequences are credible. Moreover, when compared with previously characterized single-point mutants^30^,^33^,^34^ and chimeric RuBisCO enzyme complexes^35^, our ancestral RuBisCOs display superior enzyme kinetics, further supporting the validity of the predicted ancestral sequences (Fig. 7). Many of these point mutants were generated in attempts to engineer improved kinetic properties of RuBisCO^30^,^33^. Moreover, our ancestral RuBisCOs exhibit overall better kinetics than previously described hybrid RuBisCOs composed of LSUs and SSUs from different species^35^. This further suggests that our predicted ancestral sequences for both subunits are credible proxies for the true ancestral sequences. Likewise, both the α-MRCA and the β-MRCA ancestral RuBisCOs, which originate from two separate phylogenetic clades, gravitate towards similar biochemical properties (Fig. 7a), hinting that ancestral RuBisCOs indeed initially started within the same region of the kinetic landscape that we measured.

### Examining the evolutionary context of ancestral RuBisCO and CCM

Given their biochemical properties, extant cyanobacterial RuBisCOs could still perform photosynthesis efficiently under early Phanerozoic CO_2_ (15–20 times PAL) and O_2_ (at PAL) concentrations^11^. Thus, it has been hypothesized that CCMs were not necessary and did not evolve until ∼0.4 Gyr ago^36^. Owing to the genetic complexity and prerequisites needed to evolve the multiple components involved in CCMs (for example, transporters, carboxysomes), the factors most directly amenable to evolution in response to the drastically changing atmosphere would have been the catalytic properties of RuBisCO. Consistent with this evolutionary scenario, extant cyanobacterial RuBisCOs—which operate in concert with a CCM—display high *V*_c_ and low specificity factor values, which would complement the ability of the CCM to increase the CO_2_/O_2_ ratios within the cell. (Supplementary Table 3).

To further examine the origin of CCMs, we focused on the carboxysome, the core component of the bacterial CCM. The carboxysome is an organelle composed of an array of proteins, including RuBisCO and carbonic anhydrase, encapsulated in a protein shell that functions to increase the local CO_2_ concentration around RuBisCO^37^,^38^. Cyanobacteria encapsulate the majority of the cellular RuBisCO within α-carboxysomes or β-carboxysomes, containing Form 1A and Form 1B RuBisCO, respectively^39^.

Because RuBisCO is packed into the carboxysome through an ordered assembly pathway^39^, we utilized the unique reference point of our ancestral sequences to probe the evolutionary history of the CCM. Assembly of multi-protein complexes are under strong evolutionary pressure^40^, thus we aimed to determine whether ancestral RuBisCOs could be encapsulated within the carboxysomes of extant cyanobacteria. Because the LSU has been shown to determine holoenzyme encapsulation within the carboxysome^41^, we simultaneously examined the distribution of fluorescently tagged ancestral LSU and of the carboxysomal subunit, CcmN, fused to a spectrally distinct fluorescent tag, within the β-carboxysome of an extant cyanobacterium, *Synechococcus elongatus* PCC 7942 (*Synechococcus*; Fig. 8). All tagged ancestral RbcL subunits displayed spatially discrete fluorescent puncta across the cell, coincident with CcmN: a previously described signature of carboxysome association^39^,^42^. Our results indicate that the ancestral LSUs are able to be encapsulated; selection for this property may have enabled successful lineages to avoid extinction during the changing Proterozoic atmosphere.

**Figure 1:**
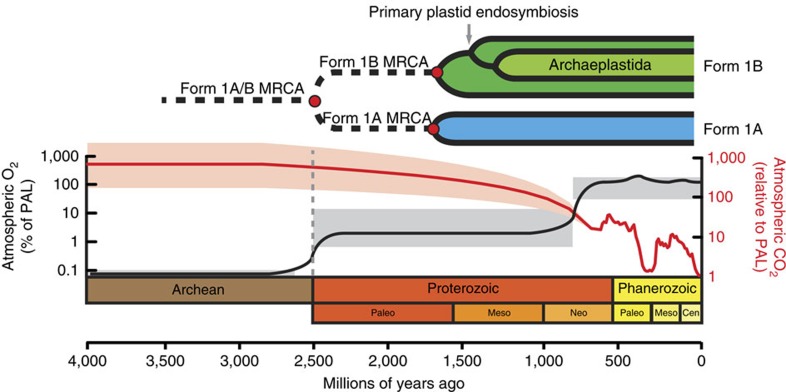
Evolution of RuBisCO and corresponding atmospheric concentrations. Both CO_2_ and O_2_ concentrations are estimates based on biogeochemical models and proxy data^8^,^9^. It is important to note that there is great uncertainty surrounding the reconstruction of atmospheric concentrations over these timescales. Estimated O_2_ concentrations (black line) over geologic time are based on the percent of PALs (%, v/v), with grey shaded boxes representing the range of possible O_2_ levels based on geological proxies. Estimated CO_2_ levels (red line) are represented in relation to present day concentrations (ratio past/present), primarily based on GEOCARB data. The red shaded area represents the extent of uncertainty surrounding the estimated Archaean and Proterozoic CO_2_ levels. The grey dashed line represents the Great Oxidation Event.

**Figure 2:**
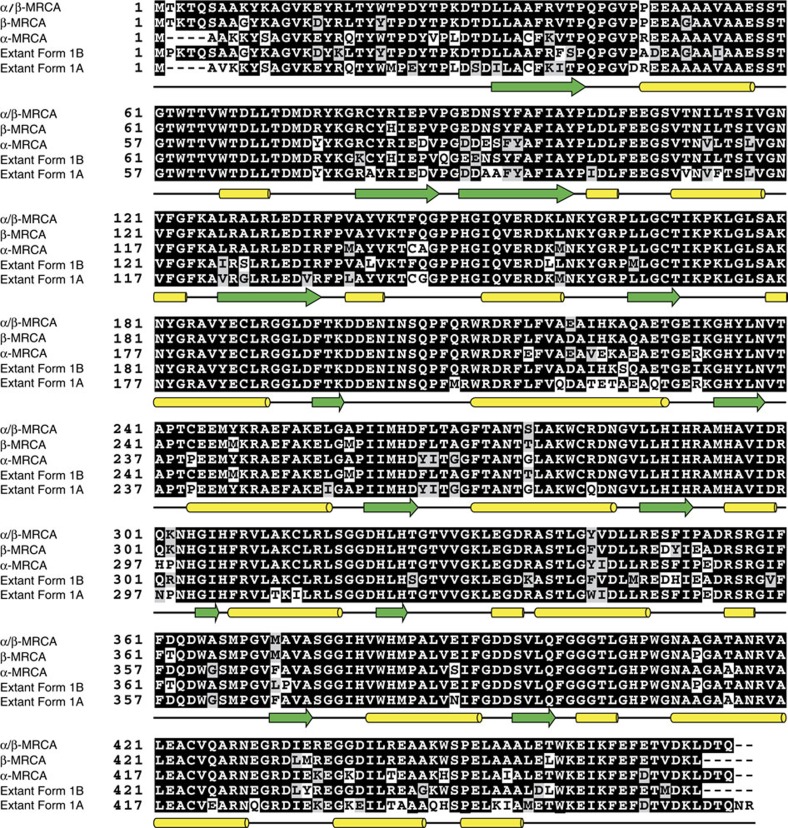
Alignment of ancestral RbcL sequences and extant Form 1A and Form 1B counterparts. Extant Form 1B sequence from *Synechococcus elongatus* PCC6301. Extant Form 1A sequence from *Halothiobacillus neapolitanus*. Residues with darker shades indicate higher conservation, while lighter shades represent lower conservation. Secondary structure based on the *Halothiobacillus neapolitanus* Form 1A RuBisCO structure, 1SVD (PDB ID), from the Protein Data Bank is denoted below as green arrows (beta sheets) and yellow tubes (alpha helices).

**Figure 3:**
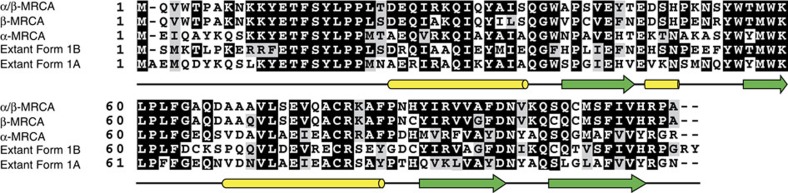
Alignment of ancestral RbcS sequences and extant Form 1A and Form 1B counterparts. Extant Form 1B sequence from *Synechococcus elongatus* PCC6301. Extant Form 1A sequence from *Halothiobacillus neapolitanus*. Residues with darker shades indicate higher conservation, while lighter shades represent lower conservation. Secondary structure based on the *Halothiobacillus neapolitanus* Form 1A RuBisCO structure, 1SVD (PDB ID), from the Protein Data Bank is denoted below as green arrows (beta sheets) and yellow tubes (alpha helices).

**Figure 4:**
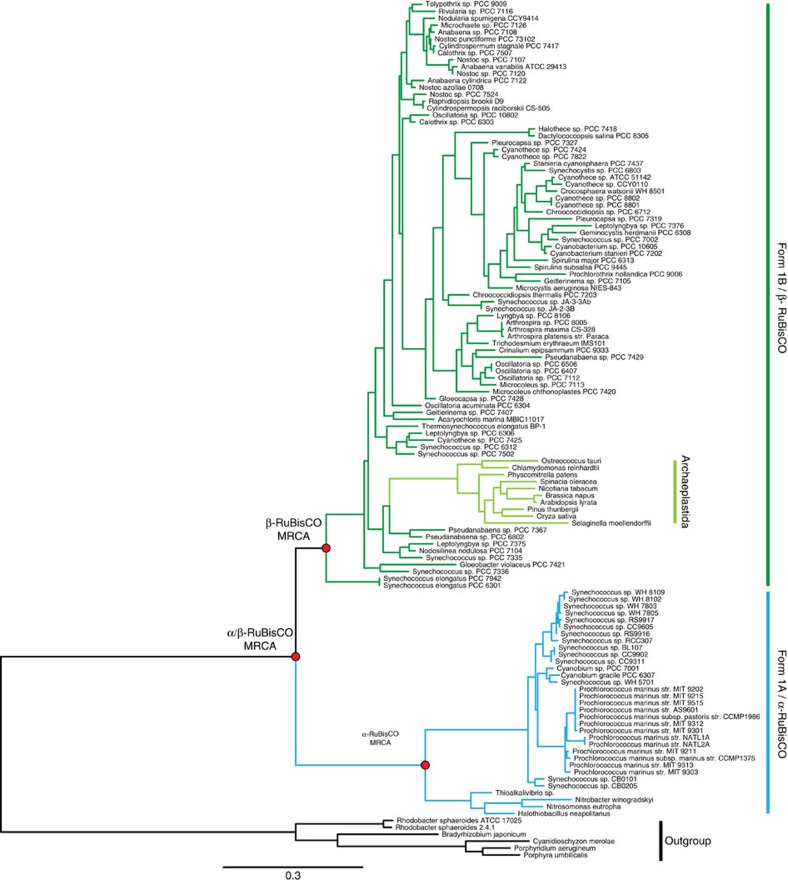
Maximum-likelihood phylogeny of RbcL. A total 125 RbcL sequences were gathered to maximize the sampling of the entire Form 1AB RuBisCO clade. Sequences were aligned with structural data using PROMALS3D. The phylogenetic tree was constructed using maximum-likelihood methods using PhyML software. The outgroup consists of Form 1C and 1D RuBisCO sequences used to root the Form 1AB subclade. The nodes of interest are shown in red, for which ancestral sequences were reconstructed. Scale bar, substitutions per site.

**Figure 5:**
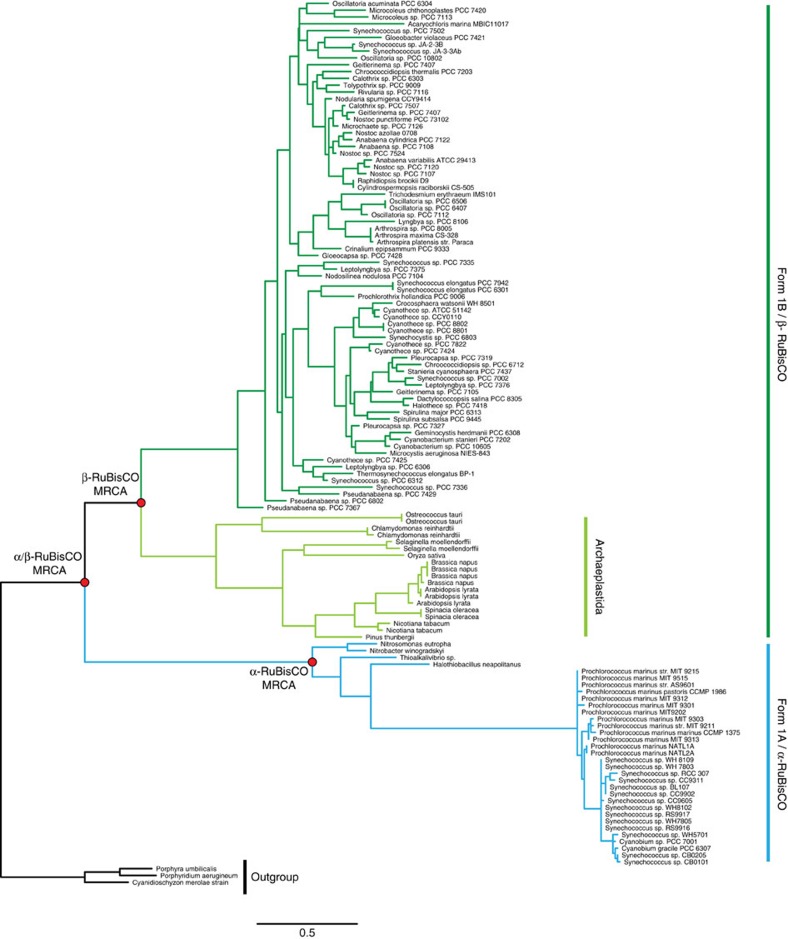
Maximum-likelihood phylogeny of RbcS. A total 131 RbcS sequences were gathered in order to maximize the sampling of the entire Form 1AB RuBisCO clade. Sequences were aligned with structural data using PROMALS3D. The phylogenetic tree was constructed using maximum-likelihood methods using PhyML software. The outgroup consists of Form 1C and 1D RuBisCO sequences used to root the Form 1AB subclade. The nodes of interest are shown in red, for which ancestral sequences were reconstructed. Scale bar, substitutions/site.

**Figure 6:**
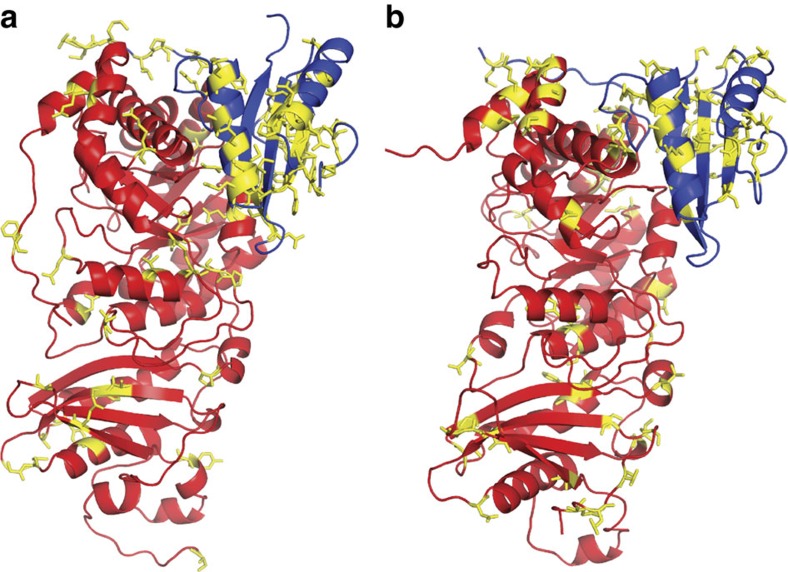
Distribution of substitutions mapped onto crystal structures of RuBisCO. (**a**) Complex of one large (red) and one small (blue) subunit from the crystal structure of Form 1B RuBisCO of *Synechococcus elongatus* PCC6301 (PDB ID: 1RBC). Substitutions between the crystal structure and the β-MRCA RuBisCO are highlighted in yellow. (**b**) Complex of one large (red) and one small (blue) subunit from the crystal structure of Form 1A RuBisCO of *Halothiobacillus neapolitanus* (PDB ID: 1SVD). Substitutions between the crystal structure and the α-MRCA RuBisCO are highlighted in yellow. The two models are similarly oriented.

**Figure 7:**
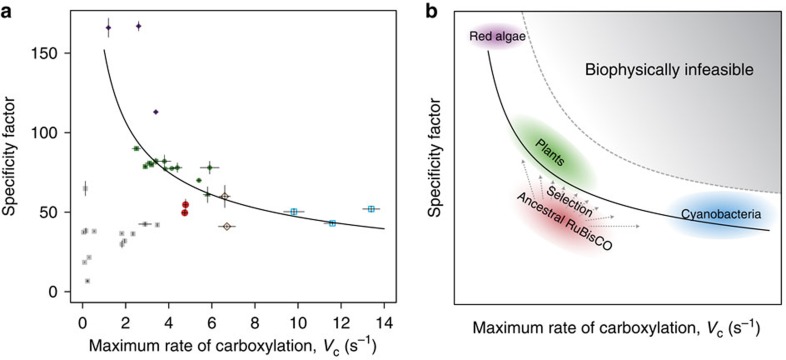
Comparison of ancestral and extant RuBisCO. (**a**) Specificity factor (*τ*) versus carboxylation rate (*V*_c_) for characterized RuBisCOs. The specificity factor is measured as the ratio of the catalytic efficiency of carboxylation to oxygenation, thus specificity factor=(*V*_c_*K*_o_)/(*V*_o_*K*_c_). The best-fit curve for extant RuBisCOs (black line) is as previously described^32^. (Form 1A (brown open diamonds); Form 1B Cyanobacteria (blue open squares); eukaryotic non-green algae (purple filled diamonds); eukaryotic green algae (green filled triangles); C4 plants (green filled circles); C3 plants (green filled squares); ancestral (red filled circles); point mutant or chimeric (grey filled squares)). Values and error bars are summarized in Supplementary Table 3, and based on 30,32,33,34,35. (**b**) Model of selective pressures pushing properties of RuBisCO towards the hypothesized protein landscape optimum—upper limits of the kinetic parameters—represented by the best-fit curve (black line). Coloured regions correspond to part **a**: ancestral (red), plants (green), eukaryotic non-green algae (purple) and cyanobacteria (blue). The grey shaded area represents the theoretical enzyme space that is biophysically infeasible for RuBisCO.

**Figure 8:**
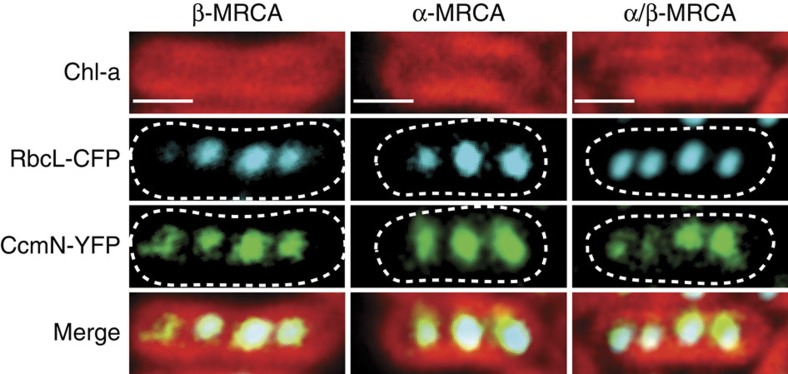
Encapsulation of ancestral RuBisCO in carboxysomes of extant cyanobacteria. Fluorescence microscopy of *Synechococcus elongatus* PCC 7942 mutants transformed with ancestral β-MRCA, α-MRCA and α/β-MRCA RbcL subunits fused to CFP (blue) and carboxysomal subunit, CcmN, fused to YFP (green). Ancestral RbcL colocalize to the carboxysome, based on CcmN-YFP fluorescence. Chlorophyll-*a* (Chl-*a*) fluorescence from the thylakoid membrane is shown in red. All the strains exhibit spatially distributed fluorescent puncta typical of carboxysome localization. Scale bars, 1 μm.

The conserved propensity for encapsulation among all our ancestral LSUs suggests that the other carboxysomal subunits (that is, shell and scaffolding proteins) may have originally evolved to accommodate structurally conserved features of the RbcL subunit. RuBisCO encapsulation may have been the first step towards the evolution of the carboxysome, just as RuBisCO aggregation increases the local enzyme concentration thus increasing overall carbon fixation in other CCMs (for example, algal pyrenoids^43^). After RuBisCO aggregation, the association with carbonic anhydrase was likely a subsequent step in the evolution of the carboxysome; this hypothesis is supported by the presence of distinct classes of carbonic anhydrase homologues, (β-carbonic anhydrase in α-carboxysomes and γ-carbonic anhydrase in β-carboxysomes), encoded in all core carboxysome operons^44^. Carboxysomes most likely originated in the later Phanerozoic period due to the dramatic decrease in CO_2_ and increase in O_2_ (ref. 36) (Fig. 1). Before this, the gradual atmospheric changes during the Proterozoic Era most likely provided weak selective pressure on ancestral RuBisCOs to improve their kinetic properties towards the kinetic landscape optimum, yielding a common ancestor from which all extant RuBisCOs descended. Subsequently, stronger selective pressures during the Phanerozoic may have forced CCMs, including carboxysomes, to evolve, as previously suggested^11^,^36^.

Because all Form 1 eukaryotic RuBisCOs display higher specificity factor values, one may logically conclude that they diverged before the origin of CCMs and after the primary endosymbiosis event. This scenario would suggest that plants and eukaryotes within the Archaeplastida lineage lack carboxysomes because carboxysomes arose after the primary plastid endosymbiosis, consistent with the observation that no Archaeplastidal genome has been shown to contain any carboxysomal genes.

Nonetheless, our data do not exclude the possibility of a more ancient origin for the carboxysome, as previously suggested^45^, given the ability of ancestral RuBisCOs to be encapsulated in extant carboxysomes. Alternatively, if RuBisCO had not been optimized by the time CCMs evolved, this may have altered the evolutionary paths of various forms of RuBisCO towards different optimized tradeoffs between *V*_c_ and specificity factor, pushing species with CCMs to optimize turnover rate over substrate specificity, as observed in extant cyanobacteria with carboxysomes. Thus, the possibility still exists that carboxysomes are truly old and arose before the plastid endosymbiosis event. However, because there is substantial evidence from both the fossil record and molecular clock studies for the Neo- to Mesoproterozoic origin of the primary plastid endosymbiosis event, at that time there would have been no selective pressure from the atmosphere to necessitate CCMs or carboxysomes owing to the high CO_2_ and low O_2_ levels of the earlier Proterozoic atmosphere.

## Discussion

Here, we present a cross-disciplinary study addressing the nature of the geologically and globally relevant enzyme, RuBisCO. Phylogenetic and bioinformatic analysis provided the predictive basis to synthesize and characterize the *in vitro* and *in vivo* biochemical properties of resurrected enzymes, thus allowing the results to be integrated with information gathered from the geological and fossil record.

Although ancestral sequence reconstruction methods are by no means perfect, especially on these timescales, our predicted sequences show clear differences between the biochemical space in which they reside and their extant counterparts. It is impossible to obtain the exact amino acid sequence of these ancestral proteins due to bioinformatic imprecision; nevertheless, these studies are invaluable to understanding the larger trends in the evolutionary trajectory of a protein family^46^,^47^. Even with a perfect sampling of all existing extant sequences, the empirically derived models used to reconstruct the ancestral sequences are unlikely to be perfect. However, future efforts to characterize the ^13^C/^12^C isotope discrimination of ancestral RuBisCOs may allow testing against the C isotope data from the geological record. Isotopic compositions have long been used to infer the contribution of various metabolic pathways and enzymes to the geochemical composition of ancient biospheres^48^. These studies are based on the uniformitarian assumption that discrimination values displayed by extant enzymes are similar to those in the geological past^49^. The isotope ratios of sediments that have long been believed to contain the isotopic signature of ancient forms of photosynthetic metabolism are already known^49^. It will be interesting to see whether data from the ancestral RuBisCOs will corroborate these isotope ratios, thus confirming the uniformitarian assumption. However, given that the measured kinetics of ancestral RuBisCOs differ from extant enzymes, it would be surprising if these proteins fractionated to the same degree as their modern counterparts.

Efforts to engineer and improve on RuBisCO have been largely unsuccessful^31^. A mechanistic model has been proposed to explain how extant RuBisCO may already be optimized, based on the observation that in optimizing specificity for CO_2_ versus O_2_ the carboxyketone product is bound more tightly to the enzyme, effectively slowing it down^29^. Thus, oxygenase activity may not be able to be eliminated due to this biophysical constraint, and engineering efforts may be more fruitful if focused on engineering CCMs that increased the local concentration of CO_2_ surrounding RuBisCO, to levels resembling the environment in which the ancestral enzymes originally evolved. In addition to the complicated and mysterious evolutionary relevance of RuBisCO's oxygenase activity, there is the possibility that it was necessary and important to introduce photorespiration. Although photorespiration is considered by many to be a wasteful process, its physiological role is still controversial, as intermediates and byproducts of the photorespiratory pathway play roles in many other metabolic processes^50^. By more thoroughly investigating the evolutionary history of RuBisCO, we may begin to piece together the biochemical underpinnings surrounding the evolution of such a globally important enzyme. Furthermore, at a biochemical and structural level, these sorts of studies may reveal the possibility that extant RuBisCOs may be stalled in a local optimum of the protein fitness landscape^51^. Finally, ancestral sequence reconstruction provides a unique platform to go back in time, opening the possibilities of forward and reverse engineering on an enzyme that has not yet been subjected to the selective pressures of history.

## Methods

### Alignment and phylogeny

A total 125 RbcL and 131 RbcS sequences spanning the Form 1 RuBisCO clade were aligned using structural information from a variety of RuBisCO crystal structures in the Protein Data Bank (PDB IDs: 1SVD, 1IR1, 1GK8, 1RBL) using the PROMALS3D^52^. Maximum-likelihood phylogenies were generated using PhyML^53^ with 100 bootstrap replicates. The LG amino-acid substitution model was chosen based on ProtTest^54^ with gamma-distributed variation (four categories) and estimation of a proportion of variable sites. The tree was rooted to the Form 1C and 1D monophyletic subclade.

### Ancestral sequence reconstruction

Maximum-likelihood methods implemented in PAML were used to resurrect ancestral sequences^23^. Sequences at internal nodes of interest (α-MRCA, β-MRCA and α/β-MRCA) from both the RbcL and RbcS phylogeny were inferred by maximum likelihood using CodeML within the PAML package. The alignment and the maximum-likelihood phylogenies generated by PhyML were used as input files for CodeML. PAML utilizes a discrete-gamma model, and sequences were reconstructed with the LG substitution model, based on ProtTest results. The α parameter was fixed with the default parameter of 0. Posterior probabilities were calculated for all amino-acid residues across the sequence, and the residue with the highest probability was assigned to each site. Predicted ancestral sequences are the same length as the alignment used; this may result in ancestral sequences that are longer than they should be. To address this issue, Hall^55^ describes a method used to estimate the positions of ancestral gaps owing to insertions and deletions, where gaps were coded as ‘g' and amino acids were coded as ‘a' to predict the inclusion or exclusion of certain positions. The ancestral sequence reconstruction of insertions and deletions was implemented using BaseML of the PAML package, using the JC69 model and maximum-likelihood phylogenetic trees used above as input files. Positions that were predicted to be ancestral gaps were omitted from the final ancestrally reconstructed sequences, as described by Hall^55^.

### Protein purification

Reconstructed sequences were synthesized and codon-optimized for expression in *E. coli* (Genscript). Corresponding RbcL and RbcS sequences were subcloned into the pET11a vector as previously described^56^. Modified pET11a vectors and pBAD33*ES*/*EL* were co-transformed into *E. coli* BL21 (DE3) cells. A previously described strain of *E*. *coli* co-transformed with pET101/D-TOPO MIT9313 cbbL/S and pGroESL was used to heterologously express the *Prochlorococcus* RuBisCO^57^. The *Synechococcus* RuBisCO was heterologously expressed as previously described^30^ using the vector pAn92 in the expression strain BL21 (DE3). Protein expression was performed as previously described^56^. Assays for the determination of the *V*_max_ and *K*_M_ for the carboxylase (*V*_c_ and *K*_c_, respectively) and of the *K*_M_ for the oxygenase (*K*_o_) activities were accompanied by controls (Supplementary Fig. 3) to establish (a) the dependence of the measured acid-stable ^14^C on the presence of RuBP; (b) the abolition of this activity by pretreatment of the extract with an excess of CABP; and (c) that products of the carboxylase activity are not themselves the cause of acid-stable ^14^C (for example, through the formation of a C4 acid by combination of ^14^CO_2_ with a C3 acid).

### RuBisCO activity assay

For determination of the *V*_max_ and *K*_M_ for the carboxylase (*V*_c_ and *K*_c_, respectively) and of the *K*_M_ for the oxygenase (*K*_o_) activities, preparations of extant cyanobacterial and ancestral α-MRCA and β-MRCA RuBisCO were rapidly extracted from *E.coli* cultures at 0 °C by sonication, followed by debris removal by centrifugation and passage through PD-10 (Sephadex G-25) columns (GE Healthcare) pre-equilibrated with 0.1 M Bicine–NaOH pH 8.0, 10 mM MgCl_2_, 1 mM EDTA, 1 mM ɛ-aminocaproic acid, 1 mM benzamidine, 1 mM KH_2_PO_4_, 2% (w/v) PEG-4000, 10 mM NaHCO_3_ and 5 mM DTT. Fractions (0.5 ml) with the most protein were combined (1.0–1.5 ml in total) and frozen/stored immediately in liquid nitrogen, until assayed. These assays were accompanied by controls (Supplementary Fig. 3) to establish (a) the dependence of the measured acid-stable ^14^C on the presence of RuBP; (b) the abolition of this activity by pretreatment of the extract with an excess of CABP; and (c) that products of the carboxylase activity are not themselves the cause of acid-stable ^14^C (for example, through the formation of a C4 acid by combination of ^14^CO_2_ with a C3 acid).

For determination of specificity factor, purer, more concentrated preparations of extant cyanobacterial and ancestral α-MRCA and β-MRCA RuBisCO were necessary, necessitating prior purification on a larger scale than necessary for the kinetic determinations. All steps in the purification process were conducted at 0–4 °C. The harvested cultures expressing all forms of RuBisCO were sonicated and clarified by centrifugation, as before. PEG-4000 and MgCl_2_ were added to the resulting supernatants giving final concentrations of 20% (w/v) and 20 mM, respectively. After 30 min at 0 °C, the PEG/MgCl_2_ precipitated RuBisCO was sedimented by centrifugation (20 min at 22,095*g*) and the pellet resuspended in 25 mM triethanolamine (pH 7.8, HCl), 5 mM MgCl_2_, 0.5 mM EDTA 1 mM ɛ-aminocaproic acid, 1 mM benzamidine, 12.5% (v/v) glycerol, 2 mM DTT and 5 mM NaHCO_3_. This material was subjected to anion-exchange chromatography using 5 ml HiTrap Q columns (GE Healthcare) pre-equilibrated with the same buffer. RuBisCO was eluted with a 0–600 mM NaCl gradient in the same buffer. Fractions containing the most RuBisCO activity (as judged by RuBP-dependent ^14^CO_2_ assimilation) were further purified and desalted by size-exclusion chromatography using a 20 cm × 2.6 cm diameter column of Sephacryl S-200 HR (GE Healthcare) pre-equilibrated and developed with (50 mM Bicine–NaOH pH 8, 20 mM MgCl_2_, 0.2 mM EDTA, 2 mM DTT). The resulting protein peak was concentrated by ultrafiltration using 20 ml capacity/150 kDa cut-off centrifugal concentrators (Thermo Pierce). The quality of preparations was verified by SDS–polyacrylamide gel electrophoresis (Supplementary Fig. S7) and confirmed by western blot using antisera raised against wheat RuBisCO.

Measurement of *V*_c_, *K*_c_ and *K*_o_ in solutions equilibrated with nitrogen, oxygen, air (79% N_2_, 21% O_2_) and 40% N_2_, 60% O_2_, were as described elsewhere^58^. After careful thawing, RuBisCO preparations were kept at 0 °C for 60 min to ensure full activity, after which 1-min assays were conducted in glass scintillation vials, fitted with caps and silicone rubber septa, in a final volume of 1 ml at 25 °C. At each O_2_ concentration, six different concentrations of bicarbonate were used, chosen to provide CO_2_ (aq) between 5 and 550 μM, each with a specific radioactivity of 3.7 × 10^10^ Bq mol^−1^ and containing 375 nmols RuBP and 25 μl of the protein extract. Values of *K*_o_ were calculated from the relationship:

*K*_c_ (at stated O_2_ concentration)=*K*_c_ (in N_2_) × (1+([O_2_]/*K*_o_)). Estimates of *V*_o_ were derived from the equation, *τ*=[*V*_c_/*K*_c_]/[*V*_o_/*K*_o_], the specificity factors (*τ*) having been determined separately (see below). The concentration of RuBisCO active sites was determined using [^14^C]CABP^59^.

The specificity factor for α-MRCA and β-MRCA RuBisCO was determined at 25 °C by the total consumption of RuBP in an oxygen electrode, as described previously^60^. A series of assays containing pure wheat RuBisCO were interspersed with those containing the ancestral RuBisCO, and the results normalized to the average value obtained from wheat RuBisCO (100.0 at 25 °C). Comparisons between this kinetic data and that of extant forms of RuBisCO are summarized in Supplementary Table 3^25^,^26^,^27^,^28^.

### Synechococccus strains

All constructs were cloned using BioBrick Assembly standard 21 (BglBrick assembly) format^61^ in *E. coli* and subsequently cloned into neutral site vector pAM1573PMS for genomic integration into the *Synechococcus* genome at Neutral Site 2. pAM1573PMS was modified from pAM1573 (ref. 62) to be BglBrick compatible. Ancestral RbcL sequences were fused to a carboxy (C)-terminal cerulean fluorescent protein (CFP) and constitutively expressed with the rplC promoter, as described by Savage *et al.*^42^ Wild-type *Synechococcus* was grown in BG11 medium with constant light at 30° C. Wild-type cells were transformed and selected for on BG11 plates with antibiotics. For co-localization studies, strains expressing CFP-tagged ancestral RbcL were subsequently transformed with another vector for expressing carboxysomal protein (CcmN) fused to a C-terminal enhanced yellow fluorescent protein driven with a CcmK2 promoter. This vector (pAM2314PMS) is a modified version of a BglBrick-modified version of pAM2314 (ref. 62) and mediates genomic integration into the *Synechococcus* genome at Neutral Site 1. Supplementary Table 4 describes the various strains used in this study.

### Fluorescence microscopy

Cells grown on solid BG11 media were spotted on to 1% agarose pads (w/v in BG11) in a 16-well chamber slide (Lab-Tek, Scotts Valley, CA) and covered with a 0.17 mm coverglass. Images were acquired on a Zeiss LSM 710 inverted confocal microscope (Carl Zeiss Inc, Thornwood, NY) using laser lines at 405, 514 and 633 nm and a × 63/1.4 NA oil-immersion objective. Images were captured using Zen 2010 (Carl Zeiss, Inc.) and analysed using ImageJ^63^.

## Additional information

**How to cite this article:** Shih, P. M. *et al.* Biochemical characterization of predicted Precambrian RuBisCO. *Nat. Commun.* 7:10382 doi: 10.1038/ncomms10382 (2016).

## Supplementary Material

Supplementary Information Supplementary Figures 1-3 and Supplementary Tables 1-4

## Figures and Tables

**Table 1:** Characterization of ancestral Form 1A and Form 1B RuBisCO.

**RuBisCO**	***V***_**c**_ **(s**^−1^**)**	***V***_**o**_ **(s**^−1^**)**	***K***_**c**_ **(μM)**	***K***_**o**_ **(μM)**	**Specificity factor (*****τ*****)**		***K***_**C**_^air^ **(μM)**	***V*****c/*****K***_**C**_^air^ **(s**^−1^**mM**^−1^**)**	**Reference**
Ancestral Form 1A	4.77±0.09	1.42±0.4	113±6	2,010±571	54.7±3.5	*n*=6	127.6	37.4	This work
Ancestral Form 1B	4.72±0.14	0.5±0.03	120±10	641±49	49.6±1.8	*n*=6	168.7	28	This work
*Prochlorococcus marinus* MIT9313 (Extant Form 1A)	6.58±0.25	0.78±0.49	309±24	1,400±300	59.9±7.0	*n*=5	366.4	18	This work
*Synechococcus* sp. PCC 6301 (Extant Form 1B)	9.78±0.48	1.58±0.17	152±23	1,231 ±135	50.3±2.0	*n*=6	184.1	53.1	This work
*Triticum aestivum* (plant)	2.92±0.08	0.91±0.03	10.9±0.9	341±33	100.0±3.8	*n*=6	19.2	152	27

Extant Form 1A and 1B are RuBisCO from *Prochlorococcus* and *Synechococcus*, respectively. Extant *K*_c_^air^ and *V*_c_/*K*_c_^air^ values were calculated as previously described^26^ from reported *K*_c_ and *K*_o_ values.
