# Supplementary material for: Biochemical characterization of predicted Precambrian RuBisCO
Source: Nat Commun. 2016 Jan 21;7:10382. doi: 10.1038/ncomms10382 (PMC4735906; doi:10.1038/ncomms10382)
Supplement: Supplementary Information — Supplementary Figures 1-3 and Supplementary Tables 1-4 [file ncomms10382-s1.pdf]

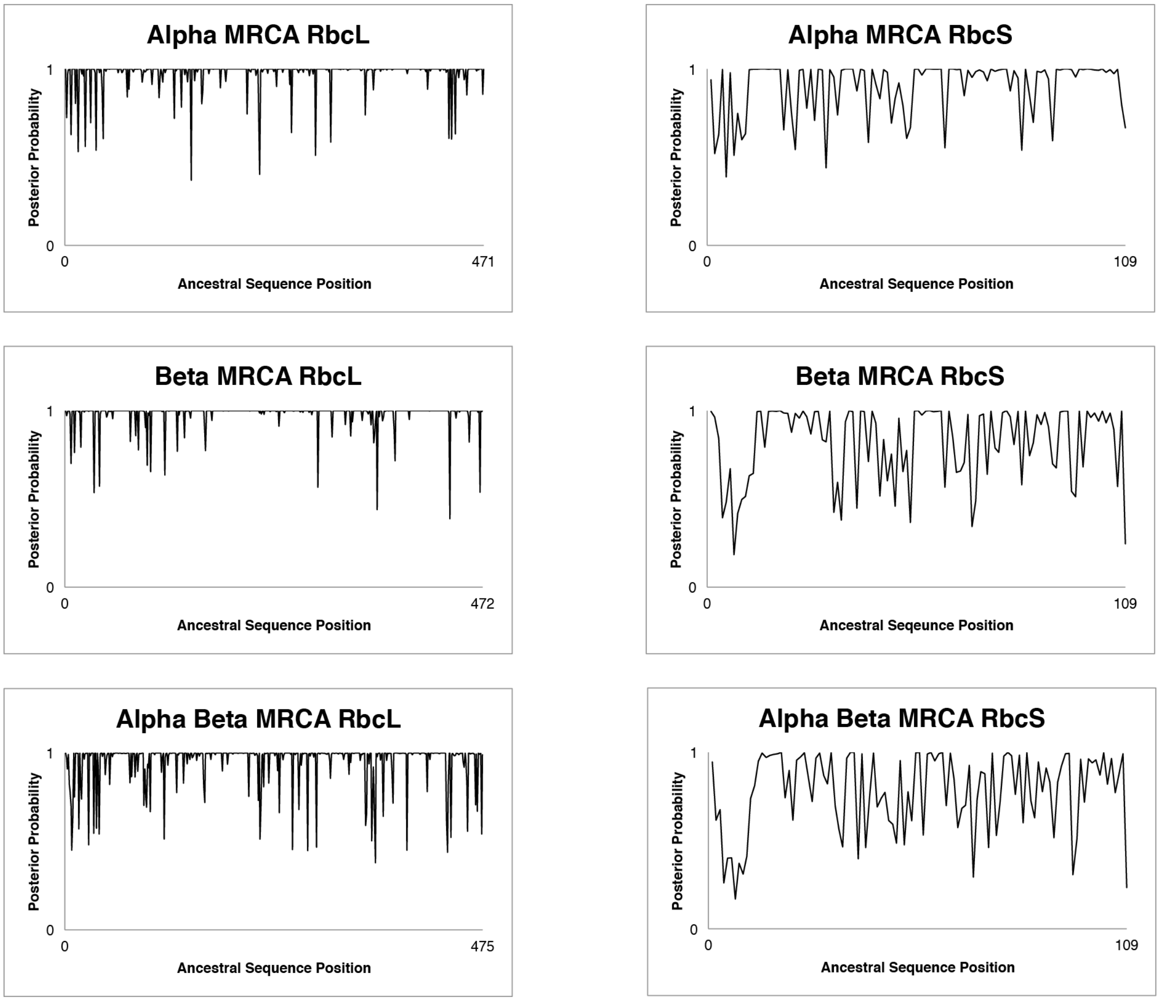

Supplementary Figure 1. Plot of posterior probabilities of ancestral RbcL and RbcS sequences by sequence position. The three internal nodes of interest for which ancestral sequences were reconstructed were the most recent common ancestor for Form 1A (Alpha MRCA), Form 1B (Beta MRCA,) and Form 1A and 1B (Alpha Beta MRCA). Posterior probabilities for each position were inferred using PAML software across the length of the sequence. The predicted amino acid at each position represents the residue with the higher posterior probability for that site.

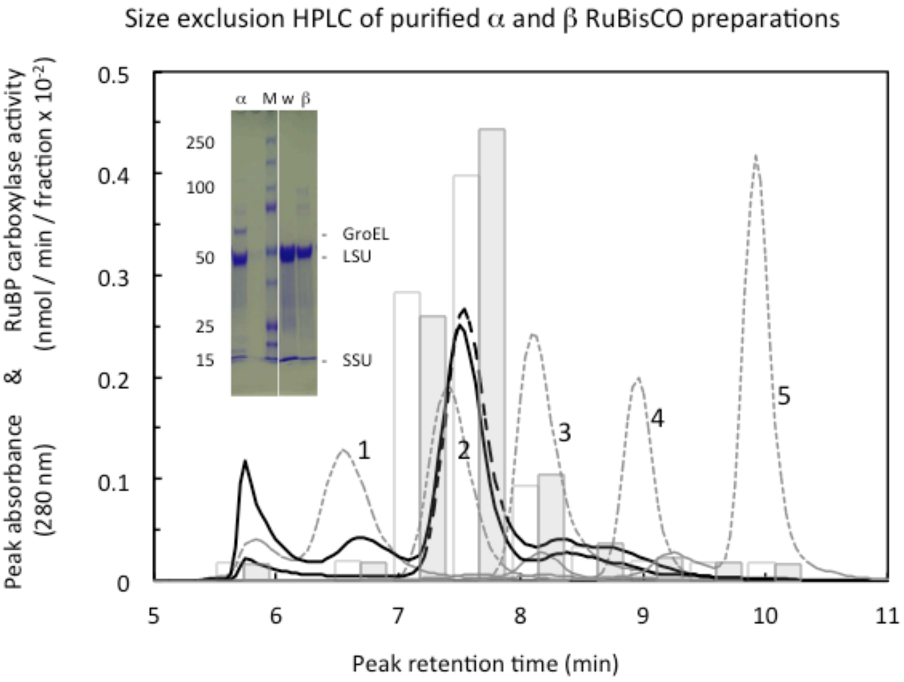

Supplementary Figure 2. Size exclusion HPLC of purified α-MRCA and β-MRCA RuBisCO. Samples of purified putative ancestral α-MRCA and β-MRCA RuBisCO were injected onto a BioSep SEC S3000 column (Phenomenex) equilibrated and developed isocratically with (0.1M sodium phosphate, pH 7.0, 0.3M NaCl) at a flow rate of 1 mL min^{-1}. The absorbance of the eluent was continuously monitored at 280nm (solid and dashed black lines) and fractions were collected at 0.5 min intervals. The RuBP dependent incorporation of $^{14}CO_2$ by 20µL aliquots of successive fractions, is shown (1A: open columns, 1B: shaded columns) although for clarity, each column only spans half the time over which it was collected. The elution profiles of a series of standard proteins, run sequentially (dashed grey lines) is also shown. 1: thyroglobulin (670 kDa); 2: wheat RuBisCO (540 kDa); 3: β-amylase (200 kDa); 4: BSA (66 kDa); and 5: carbonic anhydrase (29 kDa). Protein gel shows lanes of purified ancestral α-MRCA RuBisCO (α), wheat RuBisCO (w), and ancestral β-MRCA RuBisCO (β). Selected molecular weight markers (M) are labeled according to their mass (kDa).

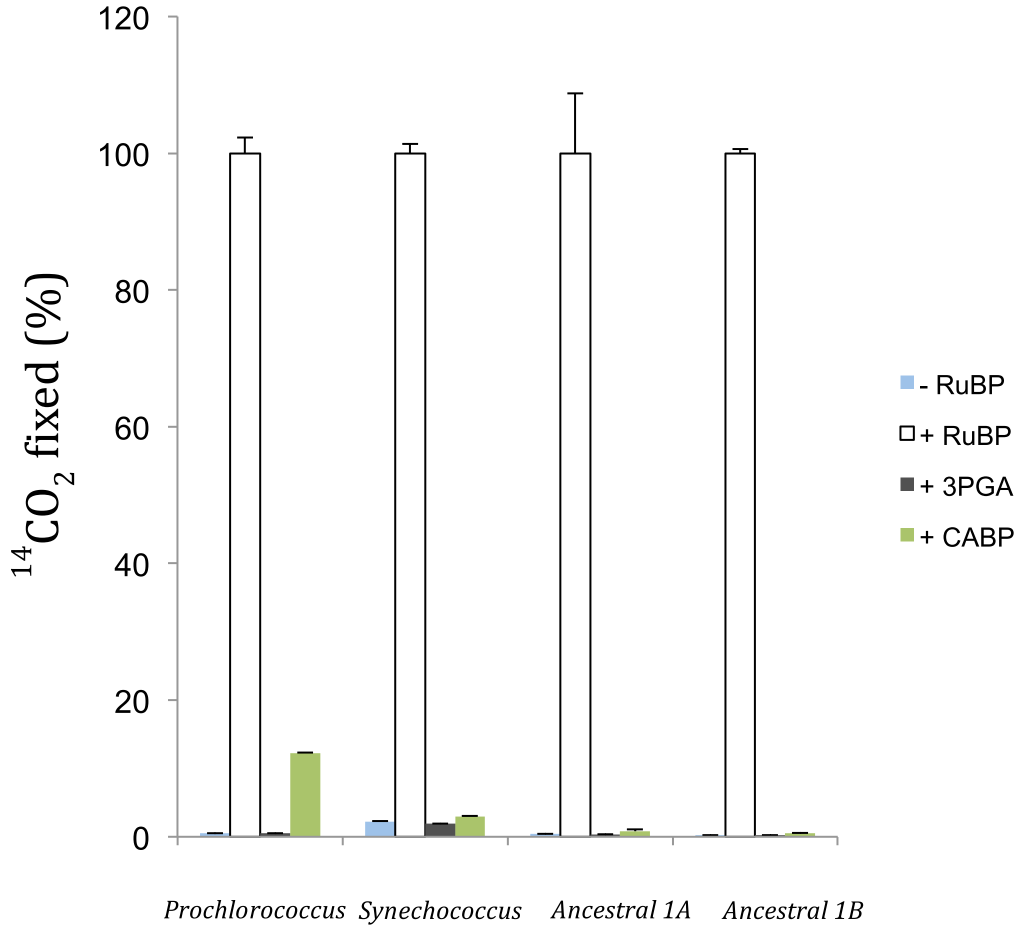

Supplementary Figure 3. The RuBP-dependence and CABP-sensitivity of the carboxylase activity of protein extracts isolated from *E. coli* expressing the indicated RuBisCO isoform. Assay conditions were exactly as described for the determination of RuBisCO rate and Michaelis constants, and utilized the same protein extracts. Where applicable, the RuBP and 3-phosphoglycerate (3PGA) concentrations were 400 and 40 μM, respectively, with 16 mM bicarbonate throughout. For CABP inhibition, RuBisCO extracts were incubated with at least a 10-fold excess of CABP (with respect to the active site concentration) for 60 min at 0°C before assay. Values are the means and standard deviations of duplicate assays. *Evidently, the measured activity was RuBP (and therefore* RuBisCO*) dependent, absent when RuBP was replaced by 3PGA and strongly inhibited by CABP: > 99% inhibition in the case of the two ancestral forms of* RuBisCO *, > 97% for Synechococcus, although only 88% inhibited in the case of Prochlorococcus* RuBisCO*. This latter situation may have led to a modest underestimation of the Rubisco concentration and therefore a corresponding overestimation of the activity of Prochlorococcus* RuBisCO .

Supplementary Table 1. GenBank GI numbers for RbcL protein sequences used in this study

<u>Organism Name</u>	<u>GenBank: GI Number</u>
**<u>Eukaryotic Form 1B</u>**	
Nicotiana tabacum	11465965
Oryza sativa	11466795
Arabidopsis lyrata	297840935
Brassica napus	383930435
Spinacia oleracea	11497536
Pinus thunbergii	7524657
Selaginella moellendorffii	255961313
Physcomitrella patens	34501407
Chlamydomonas reinhardtii	41179049
Ostreococcus tauri	113170470
**<u>Cyanobacterial Form 1B</u>**	
Geminocystis herdmanii PCC 6308	515865619
Synechococcus sp. PCC 7002	170078404
Cyanobacterium stanieri PCC 7202	428771961
Cyanobacterium sp. PCC 10605	428769037
Leptolyngbya sp. PCC 7376	427722180
Pleurocapsa sp. PCC 7319	518334212
Geitlerinema sp. PCC 7105	516258665
Prochlorothrix hollandica PCC 9006	516317085
Arthrospira maxima CS-328	209523851
Arthrospira platensis str. Paraca	284050452
Arthrospira sp. PCC 8005	300952163
Spirulina subsalsa PCC 9445	515875397
Cyanothece sp. PCC 7822	307152748
Microcystis aeruginosa NIES-843	166367530
Cyanothece sp. PCC 7424	218438350
Cyanothece sp. PCC 8801	218246439
Cyanothece sp. PCC 8802	257059481
Pleurocapsa sp. PCC 7327	428200745
Chroococcidiopsis sp. PCC 6712	2503110788*
Synechocystis sp. PCC 6803	16331392
Stanieria cyanosphaera PCC 7437	434396907
Spirulina major PCC 6313	2503155759*
Cyanothece sp. CCY0110	126657383
Crocosphaera watsonii WH 8501	67923332

Cyanothece sp. ATCC 51142	172038079
Pseudanabaena sp. PCC 7429	497312703
Crinalium epipsammum PCC 9333	428307650
Microcoleus sp. PCC 7113	428310570
Oscillatoria sp. PCC 6407	2502937509*
Oscillatoria sp. PCC 7112	428320545
Oscillatoria sp. PCC 6506	494597183
Microcoleus chthonoplastes PCC 7420	254409643
Trichodesmium erythraeum IMS101	113477810
Lyngbya sp. PCC 8106	119489316
Pseudanabaena sp. PCC 6802	518331021
Pseudanabaena sp. PCC 7367	428217181
Synechococcus sp. PCC 7502	428221690
Synechococcus sp. PCC 6312	427713058
Cyanothece sp. PCC 7425	220908796
Leptolyngbya sp. PCC 6306	515858240
Thermosynechococcus elongatus BP-1	22299049
Cylindrospermopsis raciborskii CS-505	282901108
Nostoc azollae 0708	298491113
Raphidiopsis brookii D9	282896973
Anabaena sp. PCC 7108	515516157
Calothrix sp. PCC 6303	428299658
Microchaete sp. PCC 7126	516251494
Calothrix sp. PCC 7507	427715659
Anabaena cylindrica PCC 7122	440679759
Nostoc sp. PCC 7524	427728373
Oscillatoria sp. PCC 10802	516326708
Cylindrospermum stagnale PCC 7417	434404083
Rivularia sp. PCC 7116	427736926
Tolypothrix sp. PCC 9009	2504926032*
Nostoc sp. PCC 7120	17229016
Nodularia spumigena CCY9414	119508827
Nostoc punctiforme PCC 73102	186684317
Anabaena variabilis ATCC 29413	75910111
Nostoc sp. PCC 7107	427707248
Geitlerinema sp. PCC 7407	428227232
Gloeocapsa sp. PCC 7428	434391829
Oscillatoria acuminata PCC 6304	428211462
Leptolyngbya sp. PCC 7375	493563977
Nodosilinea nodulosa PCC 7104	2509775367*
Synechococcus sp. PCC 7335	254424026
Synechococcus sp. PCC 7336	515896783
Acaryochloris marina MBIC11017	158334948

Chroococcidiopsis thermalis PCC 7203	428210196
Synechococcus elongatus PCC 6301	56750139
Synechococcus elongatus PCC 7942	81300235
Gloeobacter violaceus PCC 7421	37521725
Dactylococcopsis salina PCC 8305	428779999
Halothece sp. PCC 7418	428778075
**Bacterial Form 1A**	
Synechococcus sp. JA-2-3B'a(2-13)	86610012
Synechococcus sp. JA-3-3Ab	86605880
Halothiobacillus neapolitanus c2	261855529
Thioalkalivibrio sp. K90mix	289208043
Nitrosomonas eutropha C91	114330814
Nitrobacter winogradskyi Nb-255	75676177
Synechococcus sp. CB0205	317969561
Synechococcus sp. CB0101	318041000
Synechococcus sp. CC9605	78212297
Synechococcus sp. CC9311	113953353
Synechococcus sp. CC9902	78185181
Synechococcus sp. WH 8102	33866250
Synechococcus sp. RS9917	87123944
Synechococcus sp. WH 5701	87302502
Synechococcus sp. WH 7805	88807727
Synechococcus sp. BL107	116072222
Synechococcus sp. RS9916	116075359
Synechococcus sp. RCC307	148241918
Synechococcus sp. WH 7803	148239014
Cyanobium sp. PCC 7001	254431192
Synechococcus sp. WH 8109	260436486
Cyanobium gracile PCC 6307	427701354
Prochlorococcus marinus str. MIT 9312	78778935
Prochlorococcus marinus str. MIT 9313	33863475
Prochlorococcus marinus str. NATL2A	72383715
Prochlorococcus marinus subsp. marinus str. CCMP1375	33240002
Prochlorococcus marinus subsp. pastoris str. CCMP1986	33861107
Prochlorococcus marinus str. AS9601	123968141
Prochlorococcus marinus str. MIT 9301	126695914
Prochlorococcus marinus str. MIT 9303	124022517
Prochlorococcus marinus str. MIT 9515	123965849
Prochlorococcus marinus str. NATL1A	124025315
Prochlorococcus marinus str. MIT 9215	157412966

Prochlorococcus marinus str. MIT 9211	159903093
Prochlorococcus marinus str. MIT 9202	254525857
**Outgroup Form 1C & 1D**	
Bradyrhizobium japonicum USDA 110	27377696
Rhodobacter sphaeroides 2.4.1	77464859
Rhodobacter sphaeroides ATCC 17025	146278745
Porphyra umbilicalis	75295143
Porphyridium aerugineum	730477
Cyanidioschyzon merolae strain 10D	30468058

*IDs correspond to Integrated Microbial Gennome database gene identifier

Supplementary Table 2. GenBank GI numbers for RbcS protein sequences used in this study

Organism Name	Genbank: GI Number
**Eukaryotic Form 1B**	
Nicotiana tabacum	59800169
Nicotiana tabacum	30013663
Oryza sativa	115444275
Arabidopsis lyrata	297801784
Arabidopsis lyrata	297801782
Arabidopsis lyrata	297841389
Brassica napus	406727
Brassica napus	132091
Brassica napus	266891
Brassica napus	79013989
Spinacia oleracea	3914583
Spinacia oleracea	2529378
Pinus thunbergii	132150
Selaginella moellendorffii	302779908
Selaginella moellendorffii	302819794
Chlamydomonas reinhardtii	159488841
Chlamydomonas reinhardtii	132092
Ostreococcus tauri	308813858
Ostreococcus tauri	308813856
**Cyanobacterial Form 1B**	
Nodularia spumigena CCY9414	119508825
Tolypothrix sp. PCC 9009	516359995
Nostoc sp. PCC 7524	427728375
Gloeocapsa sp. PCC 7428	434391831
Anabaena cylindrica PCC 7122	440679761
Anabaena sp. PCC 7108	515516159
Nostoc azollae 0708	298491111
Microchaete sp. PCC 7126	516251492
Nostoc punctiforme PCC 73102	186684319
Calothrix sp. PCC 7507	427715657
Geitlerinema sp. PCC 7407	428227230
Chroococcidiopsis thermalis PCC 7203	428210198
Cylindrospermopsis raciborskii CS-505	493320873
Raphidiopsis brookii D9	282896971
Rivularia sp. PCC 7116	427736924
Oscillatoria acuminata PCC 6304	428211460

Calothrix sp. PCC 6303	428299656
Oscillatoria sp. PCC 10802	516326706
Synechococcus sp. PCC 7502	428221688
Oscillatoria sp. PCC 7112	428320547
Oscillatoria sp. PCC 6407	494597185
Oscillatoria sp. PCC 6506	300866367
Nostoc sp. PCC 7107	427707246
Nostoc sp. PCC 7120	17229018
Anabaena variabilis ATCC 29413	75910109
Microcoleus sp. PCC 7113	428310572
Microcoleus chthonoplastes PCC 7420	254409787
Synechococcus sp. JA-3-3Ab	86605882
Synechococcus sp. JA-2-3B	86610010
Crinalium epipsammum PCC 9333	428307648
Arthrospira maxima CS-328	493673333
Arthrospira platensis str. Paraca	284050450
Arthrospira sp. PCC 8005	300952161
Trichodesmium erythraeum IMS101	113477808
Pseudanabaena sp. PCC 7429	497312705
Pseudanabaena sp. PCC 6802	518331019
Pseudanabaena sp. PCC 7367	428217179
Nodosilinea nodulosa PCC 7104	515867623
Leptolyngbya sp. PCC 7375	493563975
Synechococcus sp. PCC 7335	254422428
Geitlerinema sp. PCC 7407	428227230
Lyngbya sp. PCC 8106	119489314
Geminocystis herdmanii PCC 6308	515865617
Cyanobacterium stanieri PCC 7202	428771963
Cyanobacterium sp. PCC 10605	428769035
Geitlerinema sp. PCC 7105	516258667
Leptolyngbya sp. PCC 7376	427722182
Synechococcus sp. PCC 7002	170078402
Stanieria cyanosphaera PCC 7437	434396905
Pleurocapsa sp. PCC 7319	518334210
Chroococcidiopsis sp. PCC 6712	2503110786*
Pleurocapsa sp. PCC 7327	428200747
Microcystis aeruginosa NIES-843	166367528
Spirulina subsalsa PCC 9445	515875399
Spirulina major PCC 6313	2506610002*
Prochlorothrix hollandica PCC 9006	515489639
Synechocystis sp. PCC 6803	16331394
Cyanothece sp. PCC 8801	218246441
Cyanothece sp. PCC 8802	257059483

Cyanothece sp. CCY0110	126657385
Cyanothece sp. ATCC 51142	172038077
Cyanothece sp. PCC 7424	218438348
Cyanothece sp. PCC 7822	307152750
Crocosphaera watsonii WH 8501	67923334
Halothece sp. PCC 7418	428778077
Dactylococcopsis salina PCC 8305	428780001
Synechococcus sp. PCC 7336	515896781
Gloeobacter violaceus PCC 7421	37521727
Synechococcus sp. PCC 6312	427713060
Thermosynechococcus elongatus BP-1	22299047
Leptolyngbya sp. PCC 6306	515858238
Cyanothece sp. PCC 7425	220908794
Acaryochloris marina MBIC11017	158334946
Synechococcus elongatus PCC 6301	56750138
Synechococcus elongatus PCC 7942	81300236
**Bacterial Form 1A**	
Nitrobacter winogradskyi Nb-255	75677109
Nitrosomonas eutropha C91	114330815
Thioalkalivibrio sp. K90mix	289208044
Halothiobacillus neapolitanus c2	261855528
Prochlorococcus marinus marinus CCMP 1375	33240003
Prochlorococcus marinus MIT 9303	124022518
Prochlorococcus marinus str. MIT 9211	159903094
Prochlorococcus marinus MIT9202	254525955
Prochlorococcus marinus MIT 9312	78778936
Prochlorococcus marinus MIT 9313	33863474
Prochlorococcus marinus NATL2A	72383716
Prochlorococcus marinus pastoris CCMP 1986	33861108
Prochlorococcus marinus str. AS9601	123968142
Prochlorococcus marinus MIT 9301	126695915
Prochlorococcus marinus MIT 9515	123965850
Prochlorococcus marinus NATL1A	124025316
Prochlorococcus marinus str. MIT 9215	157412967
Synechococcus sp. WH5701	87302501
Cyanobium sp. PCC 7001	254431910
Cyanobium gracile PCC 6307	427701353
Synechococcus sp. CB0101	318040999
Synechococcus sp. CB0205	317969560
Synechococcus sp. CC9311	113955247

Synechococcus sp. CC9605	78212298
Synechococcus sp. CC9902	78185180
Synechococcus sp. WH8102	33866249
Synechococcus sp. RS9917	87123945
Synechococcus sp. WH7805	88807728
Synechococcus sp. BL107	116072223
Synechococcus sp. RS9916	116075360
Synechococcus sp. RCC 307	148241919
Synechococcus sp. WH 7803	148239015
Synechococcus sp. WH 8109	260434862
**Outgroup Form 1C & 1D**	
Porphyridium aerugineum	730479
Cyanidioschyzon merolae strain 10D	30468059
Porphyra umbilicalis	34393787

*IDs correspond to IMG gene identifier

Supplementary Table 3. Kinetic parameters of various extant, mutated, chimeric, and ancestral RuBisCOs.

RuBisCO	K_c (μM)	V_c (s^{-1})	Specificity	K_o (μM)	Reference
**Form 1 A Rubisco**					
Chromatium vinosum	37 ±2	6.7 ±0.4	41 ±1	290 ±25	32
Prochlorococcus marinus MIT9313	309 ± 24	6.58 ± 0.25	59.9 ± 7.0	1,400 ± 300	this work
**Form 1B Cyanobacteria**					
Synechococcus sp. PCC7002	246 ±20	13.4 ±0.4	52 ±2	1300 ±130	32
Synechococcus sp. PCC6301	340 ±12	11.6 ±0.4	43 ±1	972 ±26	32
Synechococcus sp. PCC6301	152 ± 23	9.78 ± 0.48	50.3 ± 2.0	1,231 ±135	this work
**Form 1B Green Algae**					
Chlamydomonas reinhardtii	29 ±2	5.8 ±0.2	61 ±5	480 ±58	32
**Form 1B Non-Green Algae**					
Phaeodactylum tricornutum	27.9 ±0.4	3.4 ±0.1	113 ±1	467 ±22	32
Galdieria sulfuraria	3.3 ±0.4	1.2 ±0.1	166 ±6	374 ±92	32
Griffithsia monilis	9.3 ±0.8	2.6 ±0.1	167 ±3		32
**Form 1B C4 higher plants**					
Zea mays	34 ±2.38	4.4 ±0.22	78 ±3	810 ±97.2	32
Amaranthus hybridus	16 ±1.12	3.8 ±0.3	82 ±4	640 ±76	32
Flaveria australasica	22 ±4.7	3.84 ±0.03	77.2 ±0.3	309 ±17	32
Amaranthus edulis	18.2 ±3.6	4.14 ±0.19	77.5 ±0.2	289 ±9	32
Sorghum bicolor	30 ±0.3	5.4 ±0.12	70 ±1		32
Potulaca oleraca	13.6 ±0.1	5.9 ±0.44	78 ±4		32
**Form 1B C3 higher plants**					
Triticum aestivum	14 ±3	2.5 ±0.2	90 ±1	730 ±41	32
Spinacia oleracea	12.1 ±0.8	3.2 ±0.09	79.8 ±0.5	574 ±19	32
Nicotiana tabacum	10.7 ±0.6	3.4 ±0.1	82 ±2	295 ±71	32
Flaveria pringlei	12 ±2.1	3.11 ±0.2	80.8 ±1.2	666 ±28	32
Chenopodium alba	11.2 ±2.8	2.91 ±0.07	78.7 ±1	415 ±16	32
**Ancestral RuBisCO**					
Ancestral Form 1A	113 ±6	4.77 ±0.09	54.7 ±3.5	2010 ±571	this work
Ancestral Form 1B	120 ±10	4.72 ±0.14	49.6 ±1.8	641 ±49	this work
**Mutated/Chimeric RuBisCO**					
6301 L euk S hybrid, pVTAC223	85 ±4	0.13	64.9 ±4.6	1878 ±207	35
6301 L euk S hybrid, pANOLI	179 ±6	0.05	37.4 ±0.7	1437 ±173	35
6301 T342I	169 ±31	1.95	31.9 ±1.2	446 ±66	33
6301 T342V	111 ±13	1.83	29.8 ±2.2	364 ±44	33
6301 K339P	264 ±46	0.54	38 ±0.4	721 ±105	33
6301 A340L	185 ±12	2.34	36.4 ±1	629 ±60	33
6301 S341M	155 ±1	3.47	42 ±0.9	1428 ±305	33
Anacystis Mutant K128R		2.9 ±0.3	42.5 ±1.4		30
Anacystis Mutant K128G		0.15 ±0.01	38.3 ±1.7		30
Anacystis Mutant K128Q		0.23 ±0.05	6.7 ±0.7		30
6301 T65S	620	1.82	36.6 ±0.5		34
6301 T65A	484	0.3	21.6 ±0.2		34
6301 T65V	633	0.091	18.5 ±0.2		34

Supplementary Table 4. Strains and plasmids used in this study.

Construct	Host	Description	Reference
pAM1573	*Synechococcus*	Neutral Site 2 genomic integration vector	62
pAM1573PMS	*Synechococcus*	BglBrick modified pAM1573 vector	this work
pAM2314	*Synechococcus*	Neutral site 1 genomic integration vector	62
pAM2314PMS	*Synechococcus*	BglBrick modified pAM2314 vector	this work
PMS4622	*Synechococcus*	P_rplC::AncRbcL_α/βMRCA::CFP cloned into pAM1573PMS	this work
PMS4623	*Synechococcus*	P_rplC::AncRbcL_βMRCA::CFP cloned into pAM1573PMS	this work
PMS4624	*Synechococcus*	P_rplC::AncRbcL_αMRCA::CFP cloned into pAM1573PMS	this work
JC178	*Synechococcus*	P_ccmK2::CcmN::YFP cloned into pAM2314PMS	this work
pET11a	*E. coli*	IPTG inducible expression vector	Novagen
pET11a-AncBetaRbc	*E. coli*	IPTG inducible Ancestral β MRCA RbcL and RbcS	this work
pET11a-AncAlphaRbc	*E. coli*	IPTG inducible Ancestral α MRCA RbcL and RbcS	this work
pBAD33*ES/EL*	*E. coli*	Arabinose inducible GroEL/ES chaperone proteins	56
pET101/D-TOPO MIT9313 cbbL/S	*E. coli*	*Prochlorococcus marinus* MIT9313 RuBisCO	57
pGroESL	*E. coli*	GroEL/ES chaperone proteins	57
pAn92	*E. coli*	*Synechococcus* sp. PCC6301 RuBisCO	30
